# Effects of Combined Aerobic and Resistance Exercise on Liver Metabolomics in Mice Fed a High-Fat Diet

**DOI:** 10.3390/metabo16100761

**Published:** 2026-10-10

**Authors:** Yefan Wang, Pengpeng Gao, Zijun Hao, Lijun Wu

**Affiliations:** School of Physical Education, Shanxi University, Taiyuan 030006, China

**Keywords:** combined exercise, high-fat diet, liver metabolomics, obesity

## Abstract

**Highlights:**

**What are the main findings?**

- An 8-week combined aerobic and resistance exercise intervention significantly attenuated hepatic steatosis, lipid accumulation, and inflammation in high-fat diet-induced obese mice, even without significant body weight reduction.
- Untargeted metabolomics identified 6 potential core Differential Metabolites, with pathway enrichment analysis revealing histidine metabolism and pyrimidine metabolism linking exercise to improved hepatic lipid homeostasis and cellular repair.

**What are the implications of the main findings?**

- These findings provide metabolomic evidence that combined exercise remodels hepatic metabolic networks, offering potential mechanistic insights into how exercise protects against obesity-associated liver disease beyond weight loss alone.
- The identified MSI-validated metabolites, particularly histamine and hexanoylglycine, may serve as candidate biomarkers for monitoring exercise-induced hepatic metabolic improvements and warrant further mechanistic validation in translational studies.

**Abstract:**

Background/Objectives: Obesity is linked to hepatic metabolic dysfunction and inflammation. Combined aerobic and resistance exercise is associated with improved body composition, but its effects on liver metabolomics remain unclear. This study examined an 8-week combined exercise intervention in high-fat diet (HFD)-induced obese mice. Methods: A subset of 18 male C57BL/6J mice from a larger well-characterized cohort was divided into three groups (*n* = 6/group): standard diet control (C), HFD model (HF), and HFD with combined exercise (HFAR). The intervention comprised treadmill running (12–15 m/min, 30–60 min/day) and weighted ladder climbing (10–70% body weight load), 6 days/week. Hepatic steatosis was assessed using H&E and Oil Red O staining. Hepatic lipids, transaminases (ALT/AST), and cytokines (IL-1β, IL-6, TNF-α) were measured by ELISA. Liver metabolomics was performed via LC-MS-based untargeted profiling. Results: Despite no significant reductions in terminal body weight, fat mass, or fat percentage, HFAR mice exhibited markedly attenuated hepatic steatosis. Biochemically, exercise significantly lowered hepatic TC, TG, LDL-C, ALT, and AST (all *p* < 0.01), elevated HDL-C (*p* < 0.01), and suppressed pro-inflammatory cytokines (all *p* < 0.05). Six core metabolites (MSI Level 1a/1b) were identified based on authentic standards, highlighting histidine and pyrimidine metabolism as key hubs. Conclusions: Combined exercise was associated with attenuated hepatic pathology independent of weight loss. Integrated metabolomics revealed a signature of six core metabolites associated with this benefit, suggesting coordinated shifts across histidine, pyrimidine, purine, tryptophan, and cysteine and methionine metabolism. These findings offer valuable insights into weight-loss-independent hepatoprotection in MASLD.

## 1. Introduction

Obesity is a chronic disease that affects quality of life and life expectancy and is closely associated with the development and progression of multiple chronic diseases [1]. As the global prevalence of obesity continues to rise, excess adiposity has become a major contributor to the onset, progression, and complications of liver disease. Obesity-associated metabolic dysfunction and chronic inflammation not only increase the risk of liver disease but also impose a substantial burden on healthcare systems [2]. High-fat diet consumption, a major contributor to obesity, promotes the accumulation of triglycerides and cholesterol in the liver, resulting in increased liver weight, an elevated liver index, hepatic lipid droplet accumulation, and abnormal liver biochemical parameters [3]. These alterations may subsequently lead to hepatocellular injury and, in severe cases, liver failure [4]. Furthermore, consumption of a high-fat diet constitutes one of the principal environmental risk factors contributing to the pathogenesis and progression of metabolic dysfunction-associated steatotic liver disease (MASLD) [5].

Exercise has been shown to improve a range of obesity-related complications, with well-documented protective effects against obesity- associated liver injury [6]. Exercise interventions have been reported to reduce intrahepatic lipid content and improve liver function and metabolic parameters [7]. When selecting exercise modalities, aerobic combined with resistance training demonstrates the optimal intervention efficacy for MASLD compared with single exercise modalities [8]. As the central organ regulating carbohydrate, lipid, and protein metabolism, the liver exhibits metabolite profiles that reflect physiological and pathological metabolic states [9]. Metabolomics enables the comprehensive characterization of metabolite composition, dynamic changes, and metabolic interaction networks, thereby providing a direct assessment of the functional and phenotypic status of biological systems under specific physiological or pathological conditions [10].

While our recent study using this animal cohort revealed systemic and skeletal muscle adaptations to combined exercise [11], the liver-specific metabolic response remains poorly characterized. Given that the hepatic metabolome cannot be accurately inferred from serum or muscle data, this study performed LC-MS-based untargeted metabolomics specifically on liver tissue to identify unique hepatic metabolic reprogramming, offering independent and complementary insights into the mechanisms of exercise-induced hepatoprotection.

## 2. Materials and Methods

### 2.1. Experimental Animals and Grouping

Six-week-old male C57BL/6J mice were obtained from Beijing Vital River Laboratory Animal Technology Co., Ltd. (Beijing, China) (License No.: SYXK (Hu) 2023-0005). Following a one-week acclimatization period, the mice were randomly assigned to either a standard diet group or a high-fat diet group. Mice in the high-fat diet group were fed a high-fat diet (XTM04-001; Jiangsu Xietong Pharmaceutical Bio-engineering Co., Ltd., Nanjing, China; 5.24 kcal/g, consisting of 20% protein, 60% fat, and 20% carbohydrates as percentages of total caloric intake) for 8 weeks to induce obesity. Mice in the standard diet group received a standard chow diet (3.85 kcal/g, consisting of 24% protein, 12% fat, and 64% carbohydrates as percentages of total calories).

Successful establishment of the obesity model was defined as mean body weight in the high-fat diet group that exceeded the mean body weight of the standard diet group by at least 20% after 8 weeks of dietary intervention [12]. This study utilized a subset of animals from a larger well-characterized cohort, which included the standard diet, HF, and three different exercise modalities [11]. To specifically investigate the effects of combined exercise—which demonstrates superior efficacy for MASLD [8]—on hepatic metabolism, 18 mice were selected from this cohort and divided into three groups (*n* = 6/group): the standard diet control group (C), the high-fat diet sedentary group (HF), and the high-fat diet plus combined aerobic and resistance exercise group (HFAR). All animals were from the same batch to avoid inter-cohort variability.

All mice were housed in a specific pathogen-free (SPF) animal facility maintained at 22 ± 2 °C with a relative humidity of 50 ± 10% under a 12-h light-dark cycle and were provided ad libitum access to food and water. Mice in the C group continued to receive the standard diet, whereas mice in the HF and HFAR groups remained on the high-fat diet throughout the experimental period. All animal procedures were conducted in accordance with the guidelines of the Shanxi University Scientific Research Ethics Review Committee and were approved under approval number SXULL2025081.

### 2.2. Exercise Intervention

The exercise protocol was adapted from previously published studies with appropriate modifications [13,14]. Prior to the formal intervention, mice in the HFAR group completed a 6-day adaptation period. The aerobic adaptation protocol consisted of treadmill running (JLBehv-TDMG, Shanghai Jiliang Software Science & Technology Co., Ltd. Shanghai, China) at a speed of 9 m/min for 30 min/day. The resistance adaptation protocol consisted of unweighted ladder climbing (Lad-V-M85, Beijing Cinontech Co., Ltd. Beijing, China) using a 1-m ladder positioned at an 80° incline, with two sets per day and three climbs per set. The formal exercise intervention was conducted for 8 weeks. Mice in the HFAR group underwent exercise training 6 days per week, with aerobic training performed on Mondays, Wednesdays, and Fridays and resistance training performed on Tuesdays, Thursdays, and Saturdays. No exercise was performed on Sundays. The detailed training protocol is presented in Table 1.

### 2.3. Sample Collection and Processing

To minimize the acute effects of food intake on the hepatic metabolomic profile and to ensure a consistent metabolic baseline across all experimental groups, all mice were fasted for 24 h prior to tissue collection, while water was provided ad libitum. For the HFAR group, the last exercise session was performed 48 h before tissue collection, consisting of a 24 h rest period followed by the 24 h fasting period. This ensured a consistent washout period to eliminate the acute effects of exercise. Body weight was recorded on the day of tissue collection. General anesthesia was induced by isoflurane inhalation, and an adequate depth of anesthesia was confirmed by the absence of the plantar reflex. Mice were euthanized by cervical dislocation. Immediately after euthanasia, the liver and adipose tissues (subcutaneous, perirenal, and epididymal adipose tissues) were harvested. After excess surface moisture was removed with filter paper, the tissues were weighed. Subsequently, the liver was divided into three portions. One portion was used for frozen-section preparation, whereas the remaining portions were snap-frozen in liquid nitrogen and stored at −80 °C until further analysis. The percentage of white adipose tissue (WAT%) was calculated using the following formula: (subcutaneous fat [g] + perirenal fat [g] + epididymal fat [g])/mouse body weight (g) × 100% [15].

### 2.4. Enzyme-Linked Immunosorbent Assay (ELISA)

Commercial ELISA kits were used to determine hepatic levels of total cholesterol (TC), triglycerides (TG) low-density lipoprotein (LDL), high-density lipoprotein (HDL), alanine aminotransferase (ALT), aspartate aminotransferase (AST), and the inflammatory cytokines interleukin (IL) 1β, IL-6, and tumor necrosis factor (TNF)-α according to the manufacturer’s instructions (Wuhan Colorful Gene Biotech Co., Ltd. Wuhan, China, JYM0713Mo, JYM0190Mo, JYM0711Mo, JYM0709Mo, JYM0580Mo, JYM0576Mo, JYM0012Mo, JYM0531Mo, JYM0218Mo).

### 2.5. Histological Analysis of Liver Tissue

Livers were fixed in 4% paraformaldehyde for 24 h at 4 °C prior to processing. Frozen liver sections (8–10 μm thick [16]) were prepared for histological evaluation. Hematoxylin and eosin (H&E) staining was performed using a commercial kit (Servicebio, Wuhan, China, G1003) to assess hepatocellular morphology, lipid vacuole distribution, and inflammatory cell infiltration under light microscopy, with all evaluations conducted in a blinded manner by two independent investigators. Oil Red O staining (Servicebio, G1015) was used to evaluate hepatic lipid accumulation. The Oil Red O-positive area was quantified using HALO image analysis (Indica Labs, v3.2.1825.0): five random non-overlapping fields per section were captured at 20.0× magnification, with a fixed intensity threshold of 50–255 applied to distinguish positive staining from background signal. The extent of lipid accumulation was expressed as the percentage of the positively stained area relative to the total section area.

### 2.6. Untargeted LC-MS-Based Liver Metabolomics

Liver samples were extracted using pre-cooled methanol/water (7:3, *v*/*v*) containing [13C6]-sorbitol and [2H4]-succinic acid as internal standards. Chromatographic separation was performed on a Vanquish UHPLC system equipped (Thermo Fisher Scientific, Waltham, MA, USA) with a Waters ACQUITY Premier HSS T3 column (Waters Corporation, Milford, MA, USA) (1.8 μm, 2.1 mm × 100 mm). The mobile phase consisted of (A) 0.1% formic acid in water and (B) 0.1% formic acid in acetonitrile, delivered at a flow rate of 0.4 mL/min with a column temperature of 40 °C and an injection volume of 4 μL. The gradient elution program was as follows: 0.0 min (95% A, 5% B), 1.0 min (80% A, 20% B), 3.0 min (1% A, 99% B), 4.5 min (1% A, 99% B), 4.6 min (95% A, 5% B), and 6.0 min (95% A, 5% B). Mass spectrometric analysis was conducted on a Q Exactive HF-X (Thermo Fisher Scientific, Waltham, MA, USA) mass spectrometer equipped with a HESI source in both positive and negative ion modes (scan range m/z 75–1000). The spray voltages were set at 3500 V (ESI+) and 3200 V (ESI-). The sheath gas and auxiliary gas were set at 30 and 5 Arb, respectively. The ion transfer tube and vaporizer temperatures were maintained at 320 °C and 300 °C, respectively. Full-scan MS1 data were acquired at a resolution of 35,000 with an AGC target of 1.00 × 10^6^. MS2 spectra were acquired at a resolution of 17,500 with an AGC target of 2.00× 10^5^, collision energies of 30, 40, and 50, a signal intensity threshold of 1.00 × 10^6^, and a Top N of 10 with a dynamic exclusion duration of 3 s. Raw data were converted to mzML format using ProteoWizard (v3.0.21229) and preprocessed via XCMS (v4.11.3) for peak extraction and retention time correction. Features with >50% missing values were filtered out, and missing values were imputed using KNN (for blanks < 50%) or 1/5 of the minimum value (for blanks > 50%). Metabolites with a coefficient of variation < 30% in quality control (QC) samples were retained for subsequent analyses. To further monitor instrument stability, the stable isotope-labeled internal standards [13C6]-sorbitol and [2H4]-succinic acid were detected stably across all QC injections. The median peak area RSD for all features in QC samples was 1.35%, with 99.1% of features exhibiting RSD < 20%, indicating excellent instrument performance throughout the analytical run. Signal correction was performed via support vector regression (SVR) to correct batch effects.

Unsupervised principal component analysis (PCA) was performed using the prcomp function in R (https://www.r-project.org, accessed on 12 December 2025), with data scaled to unit variance prior to analysis. Orthogonal partial least squares-discriminant analysis (OPLS-DA) was used to discriminate group-specific metabolic features, with model robustness validated by 200-permutation testing and assessment of Q^2^ intercept values.

Differential metabolites were initially screened based on OPLS-DA variable importance in projection (VIP) > 1.0 and absolute fold change ≥ 1.2 (i.e., fold change ≥ 1.2 or ≤0.83), and nominal Student’s *t*-test *p* < 0.05 (uncorrected for FDR). To identify core metabolites associated with the exercise intervention, we further restricted the candidate list to metabolites meeting all of the following criteria: (i) present in the intersection of differential metabolites between C vs. HF and HF vs. HFAR comparisons; (ii) classified as endogenous metabolites (exogenous environmental contaminants and drug-derived compounds were excluded, as verified by HMDB/METLIN database matching); (iii) exhibiting reversed abundance trends in HF vs. HFAR relative to C vs. HF (i.e., metabolites increased by HFD but reduced by exercise, or vice versa); and (iv) for the final core metabolites, annotated to Metabolomics Standards Initiative (MSI) Level 1a (high-confidence match based on authentic standards, Total.score > 0.90) or Level 1b (moderate-confidence match based on authentic standards, 0.50 < Total.score < 0.90), based on matching retention time (RT), MS1, and MS2 spectra to authentic standards in the service provider’s in-house database (MWDB, Metware Biotechnology). The commercial sources, catalog numbers, and CAS numbers of the authentic standards for these six metabolites are provided in Supplementary Table S4, and the corresponding MS/MS mirror plots are provided in Supplementary Figure S1.

Metabolic pathway enrichment analysis was performed using MetaboAnalyst 6.0 (www.metaboanalyst.ca, accessed on 12 April 2026), with pathways considered significantly enriched at *p* < 0.05 (no FDR correction was applied at the pathway level to avoid over-conservatism in this exploratory study). The metabolomics data generated in this study have been deposited in the MetaboLights repository under accession number: MTBLS15011. Available at: https://www.ebi.ac.uk/metabolights/MTBLS15011 (accessed on 1 October 2026).

### 2.7. Statistical Analysis

Statistical analyses were performed using SPSS Statistics version 27.0 and GraphPad Prism version 10.0. Data normality was assessed using the Shapiro–Wilk test, and homogeneity of variances among groups was evaluated using Levene’s test. For data that satisfied the assumptions of normality and homogeneity of variance, one-way analysis of variance was performed, followed by Tukey’s honestly significant difference (HSD) post hoc test for multiple pairwise comparisons. Results are presented as the mean ± standard deviation (Mean ± SD). For data that did not meet the assumptions for parametric analysis, the Kruskal–Wallis test was used, followed by pairwise comparisons using the Mann–Whitney *U* test with Bonferroni correction (adjusted α = 0.005). Results are presented as the median and interquartile range (IQR). Associations between differentially expressed metabolites and hepatic parameters were analyzed using Spearman’s rank correlation analysis.

## 3. Results

### 3.1. Effects of Combined Aerobic and Resistance Exercise on Body Weight, Body Fat Percentage, and Energy Intake in Mice with High-Fat Diet-Induced Obesity

**Figure 1:**
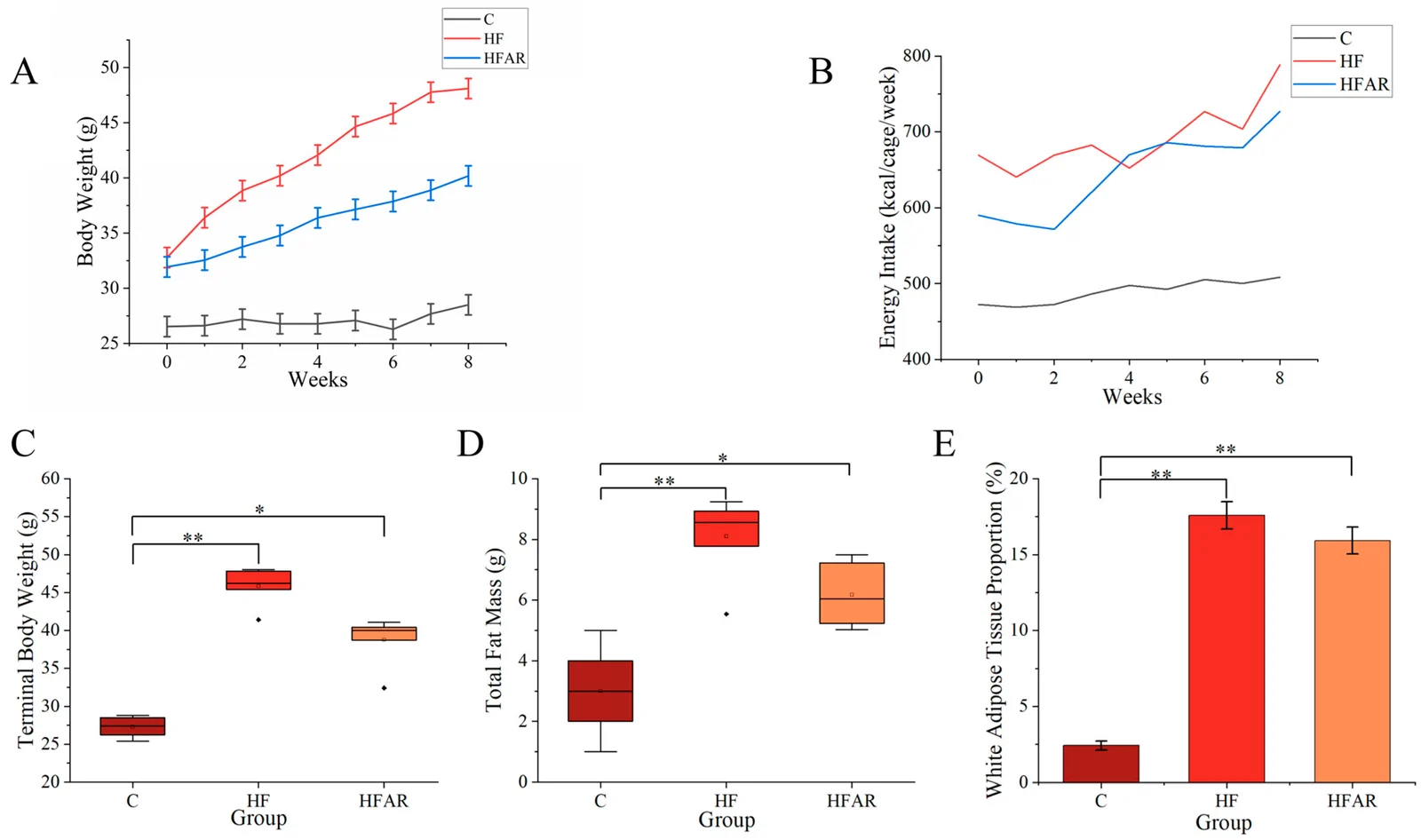
Effects of combined aerobic and resistance exercise on body weight, energy intake, and adiposity in mice. (**A**) Changes in body weight changes during the 8-week intervention period. (**B**) Weekly energy intake. (**C**) Terminal body weight at the end of the intervention. (**D**) Total fat mass. (**E**) White adipose tissue percentage (%). * *p* < 0.005, ** *p* < 0.001 vs. the HF group.

Body weight was monitored throughout the 8-week intervention period. As shown in Figure 1A, mice in Group C exhibited a gradual increase in body weight over time. In contrast, mice in the HF group showed a markedly greater increase in body weight, which remained significantly higher than that of the other groups throughout the intervention. Mice in the HFAR group exhibited a slower rate of body weight gain, with body weight values falling between those of the C and HF groups (Figure 1A). At the end of the intervention, the final body weight was significantly lower in the C group than in both the HF group (*p* < 0.001) and the HFAR group (*p* < 0.005). No statistically significant difference in the final body weight was observed between the HF and HFAR groups. Similar trends were observed in final fat mass (Figure 1D) and body fat percentage (Figure 1E).

Weekly energy intake (Figure 1B) was recorded on a per-cage basis (kcal/cage/week). The HF group consistently exhibited the highest energy intake, while the HFAR group showed a temporal pattern similar to the HF group but tended to be lower for most of the intervention period. Due to the cage-level measurement unit (*n* = 1 cage per group), no inferential statistical tests were applied to energy intake data.

### 3.2. Effects of Combined Aerobic and Resistance Exercise on Liver Weight and Histopathological Changes in Mice with a High-Fat Diet-Induced Obesity

**Figure 2:**
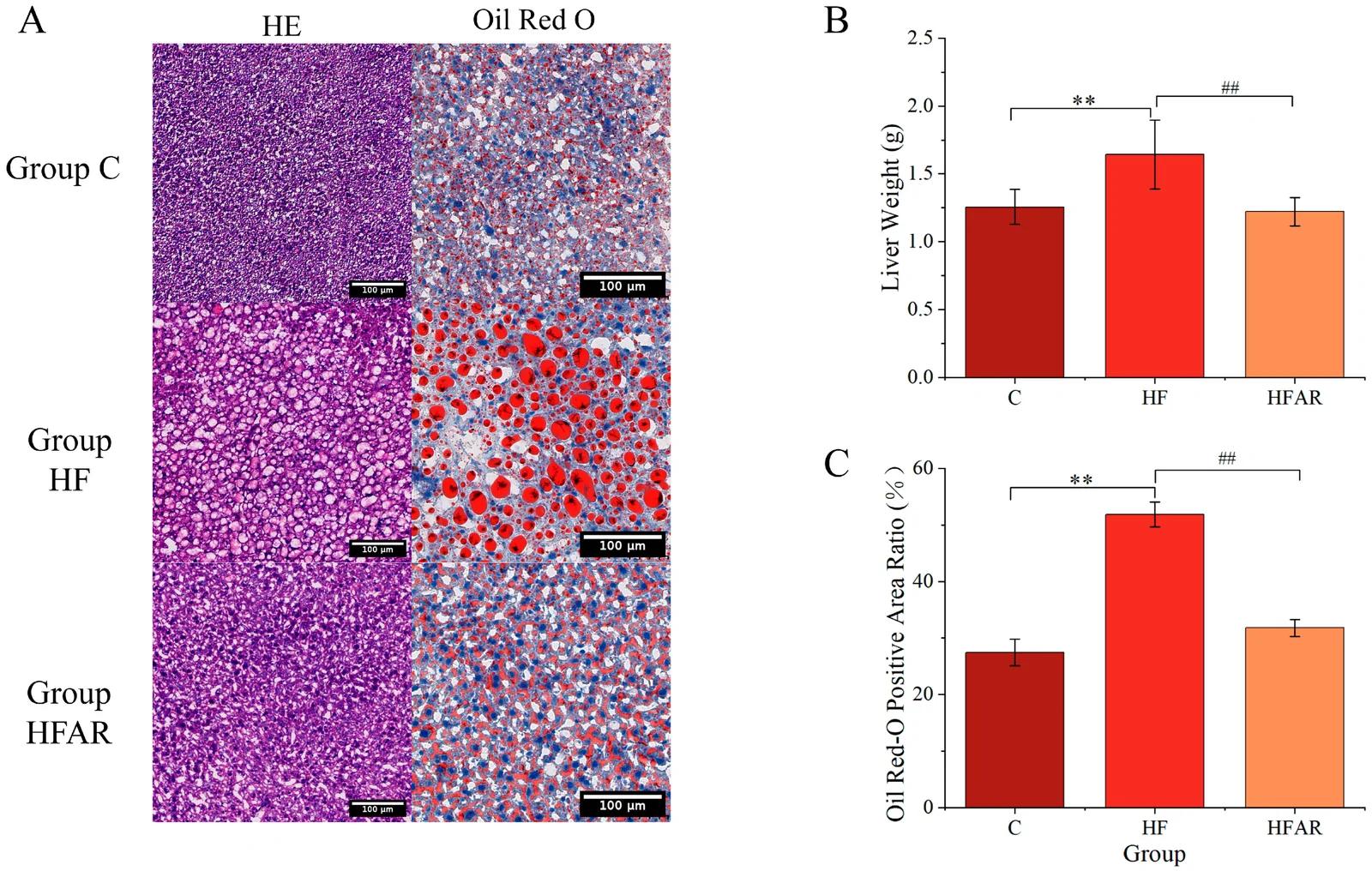
Effects of combined aerobic and resistance exercise on liver histopathology and hepatic lipid accumulation. (**A**) Representative H&E-stained and Oil Red O-stained liver sections from the control (C), high-fat diet (HF), and high-fat diet with combined aerobic and resistance exercise (HFAR) groups (scale bar = 100 μm). (**B**) Liver weight. (**C**) Quantification of the Oil Red O-positive area (%). ** *p* < 0.01 vs. the C group; ## *p* < 0.01 vs. the HF group.

Liver weights for each experimental group are presented in Figure 2B. Mice in the HF group exhibited significantly greater liver weight than those in the C group (*p* < 0.01). In contrast, liver weight was significantly lower in the HFAR group than that in the HF group (*p* < 0.05). Histopathological examination of H&E-stained liver sections (Figure 2A) demonstrated that hepatocytes in the C group were regularly arranged with preserved cellular architecture and no evident hepatic steatosis. In the HF group, hepatocytes exhibited extensive cytoplasmic lipid vacuolation, with displacement of the nuclei toward the cell periphery, consistent with hepatic steatosis. In contrast, the HFAR group showed a marked reduction in both the number and size of intracellular lipid vacuoles. Hepatocyte nuclei exhibited a more central localization, indicating an attenuation of hepatic steatosis compared with the HF group.

Oil Red O staining further confirmed these findings. Quantitative analysis (Figure 2C) demonstrated that the Oil Red O-positive area was significantly greater in the HF group than in the C group (*p* < 0.01), whereas the HFAR group exhibited a significantly lower Oil Red O-positive area than the HF group (*p* < 0.05).

### 3.3. Effects of Combined Aerobic and Resistance Exercise on Hepatic Lipid Metabolism, Liver Injury, and Inflammatory Markers in Mice with High-Fat Diet-Induced Obesity

**Figure 3:**
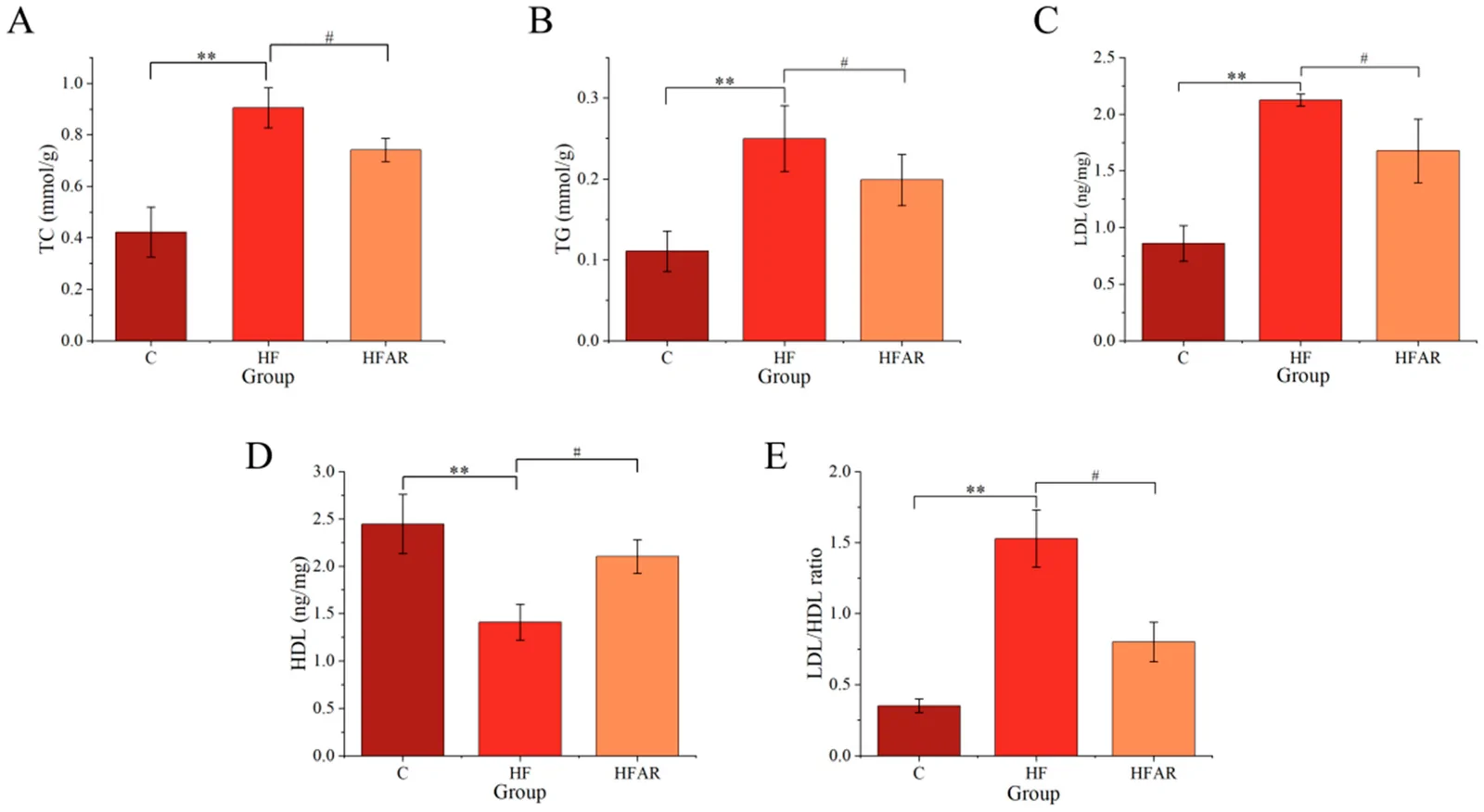
Effects of combined aerobic and resistance exercise on the hepatic lipid profile and liver injury markers. Hepatic levels of (**A**) total cholesterol (TC), (**B**) triglyceride (TG), (**C**) low-density lipoprotein cholesterol (LDL-C), (**D**) high-density lipoprotein cholesterol (HDL-C), (**E**) the LDL-cholesterol (LDL-C)/HDL-cholesterol (HDL-C) ratio in the control (C), high-fat diet (HF), and high-fat diet with combined aerobic and resistance exercise (HFAR) groups. ** *p* < 0.01 vs. the C group; # *p* < 0.01 vs. the HF group.

**Figure 4:**
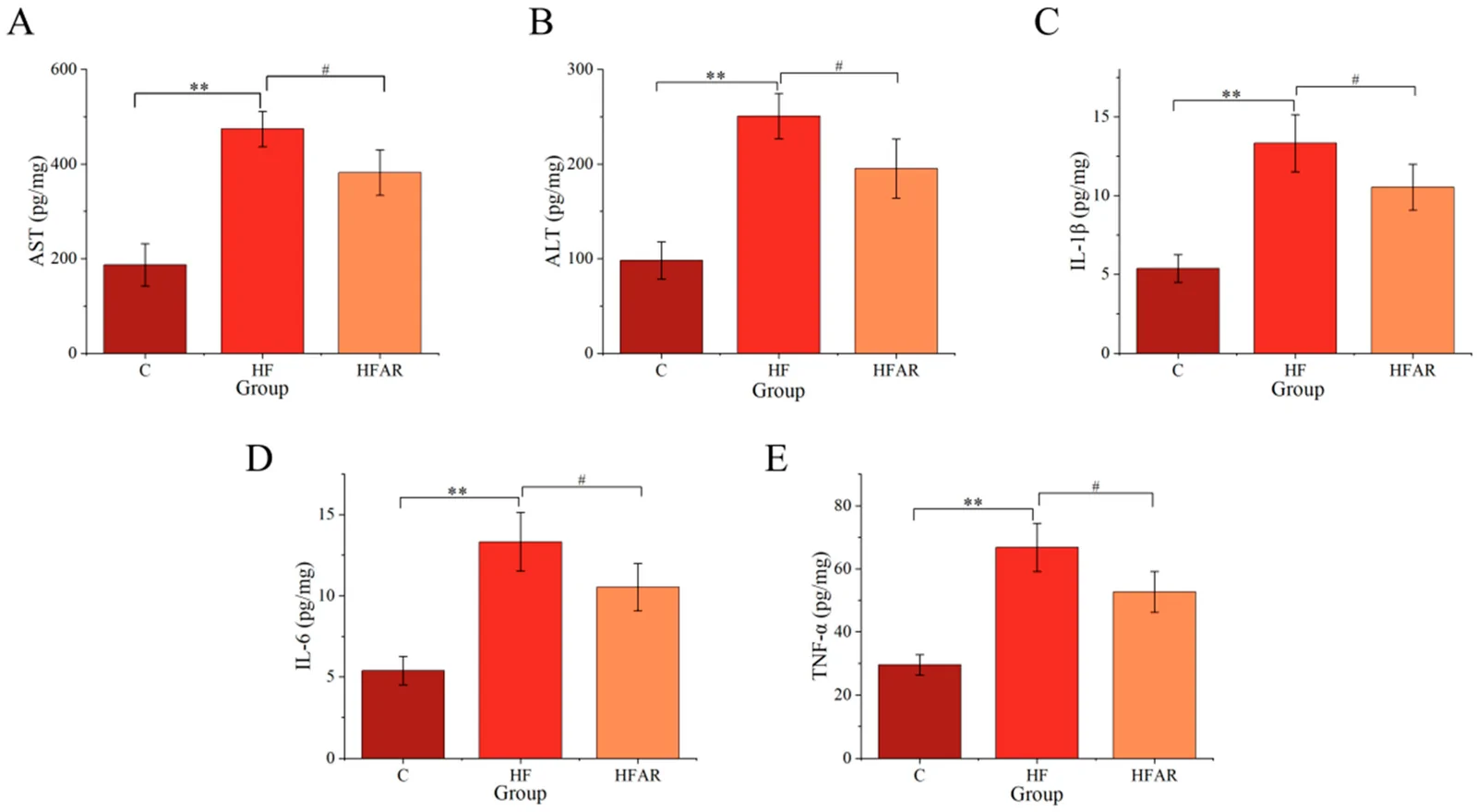
Effects of combined aerobic and resistance exercise on hepatic inflammatory cytokine levels. Hepatic levels of (**A**) Aspartate aminotransferase (AST), (**B**) Alanine aminotransferase (ALT), (**C**) Interleukin-1 beta (IL-1β), (**D**) Interleukin-6 (IL-6), and (**E**) Tumor necrosis factor-alpha (TNF-α) in the control (C), high-fat diet (HF), and high-fat diet with combined aerobic and resistance exercise (HFAR) groups. ** *p* < 0.01 vs. the C group; # *p* < 0.05 vs. the HF group.

To further evaluate the effects of combined aerobic and resistance exercise on hepatic lipid metabolism, liver injury, and inflammation, the hepatic levels of TC, TG, LDL, HDL, ALT, AST, and the inflammatory factors TNF-α, IL-6, and IL-1β were measured using ELISA. As shown in Figure 3 and Figure 4, mice in the HF group exhibited significantly higher hepatic levels of TC, TG, LDL, ALT, AST, TNF-α, IL-6, and IL-1β, as well as a significantly higher LDL-cholesterol (LDL-C)/HDL-cholesterol (HDL-C) ratio, than mice in the C group (*p* < 0.01). In contrast, hepatic HDL-C levels were significantly lower in the HF group (*p* < 0.01). Following the 8-week combined aerobic and resistance exercise intervention, mice in the HFAR group exhibited significant reductions in hepatic TC, TG, LDL, ALT, AST, TNF-α, IL-6, and IL-1β, and the LDL-C/HDL-C ratio compared with the HF group (*p* < 0.05). Hepatic HDL-C levels were significantly increased following the exercise intervention (*p* < 0.05).

### 3.4. Effects of Combined Aerobic and Resistance Exercise on Hepatic Differential Metabolites and Metabolic Pathways in Mice with High-Fat Diet-Induced Obesity

**Figure 5:**
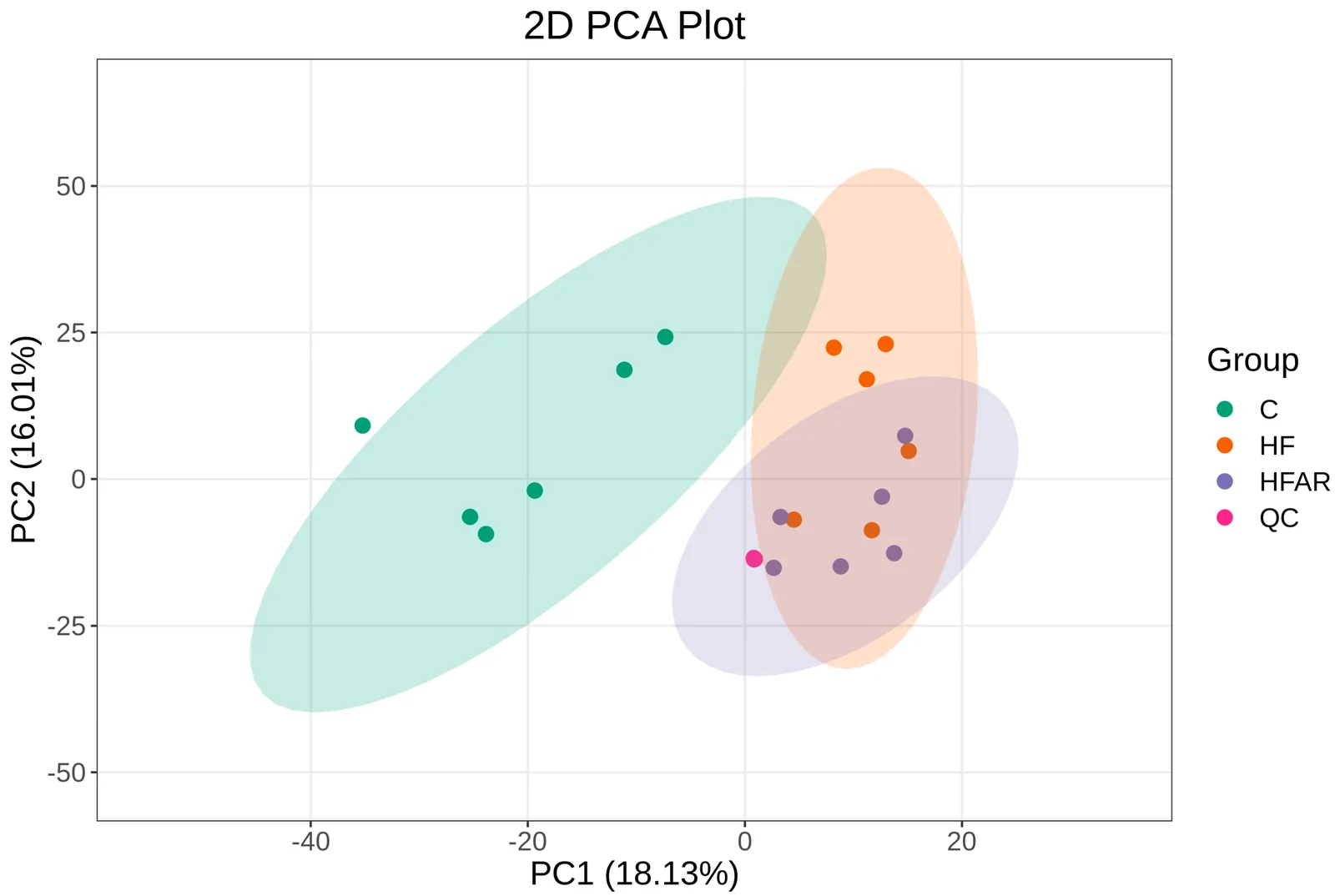
PCA score plot of hepatic metabolomic profiles from control (C, green), high-fat diet (HF, orange), and HF with combined aerobic-resistance exercise (HFAR, purple) groups. Quality control (QC) samples (pink) clustered tightly, indicating high instrument stability.

**Figure 6:**
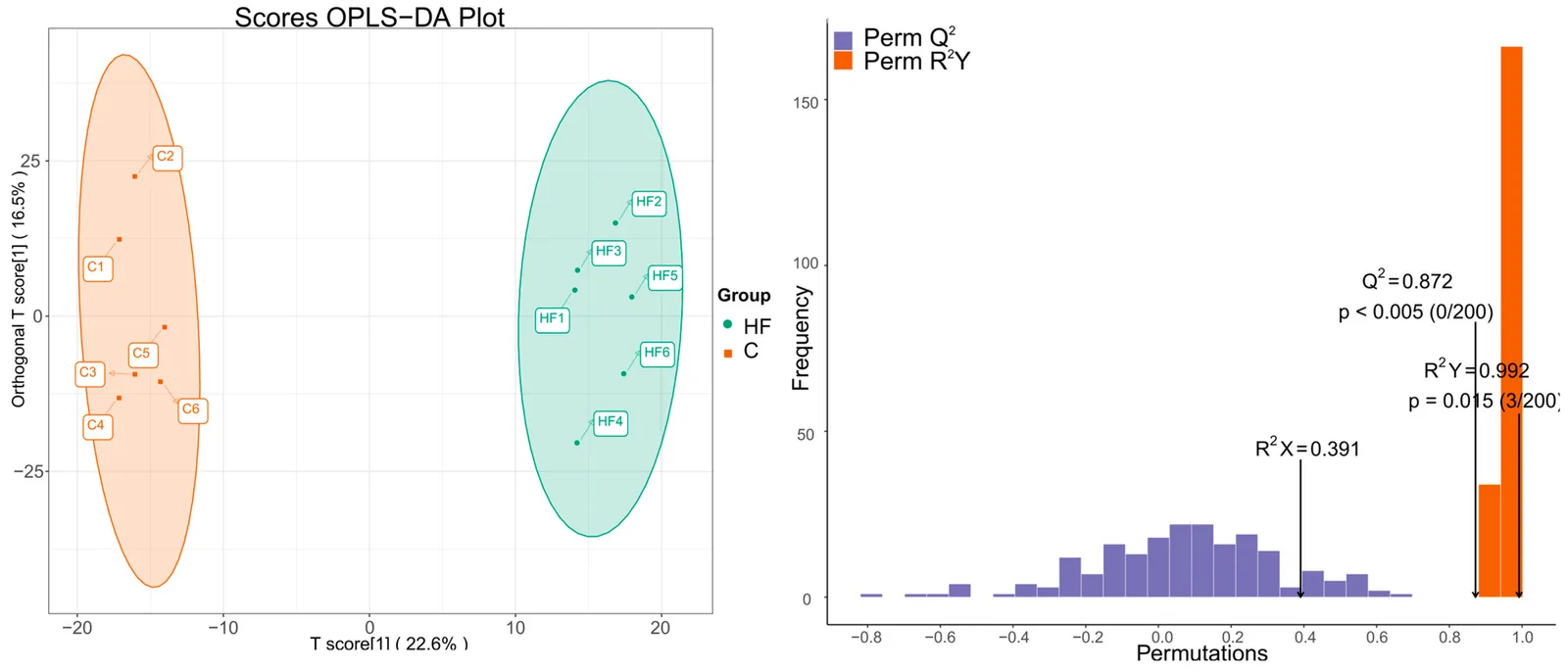
Score plot comparing the Control (C, orange) and High-Fat Diet (HF, green) groups. Permutation test for the C vs. HF model.

**Figure 7:**
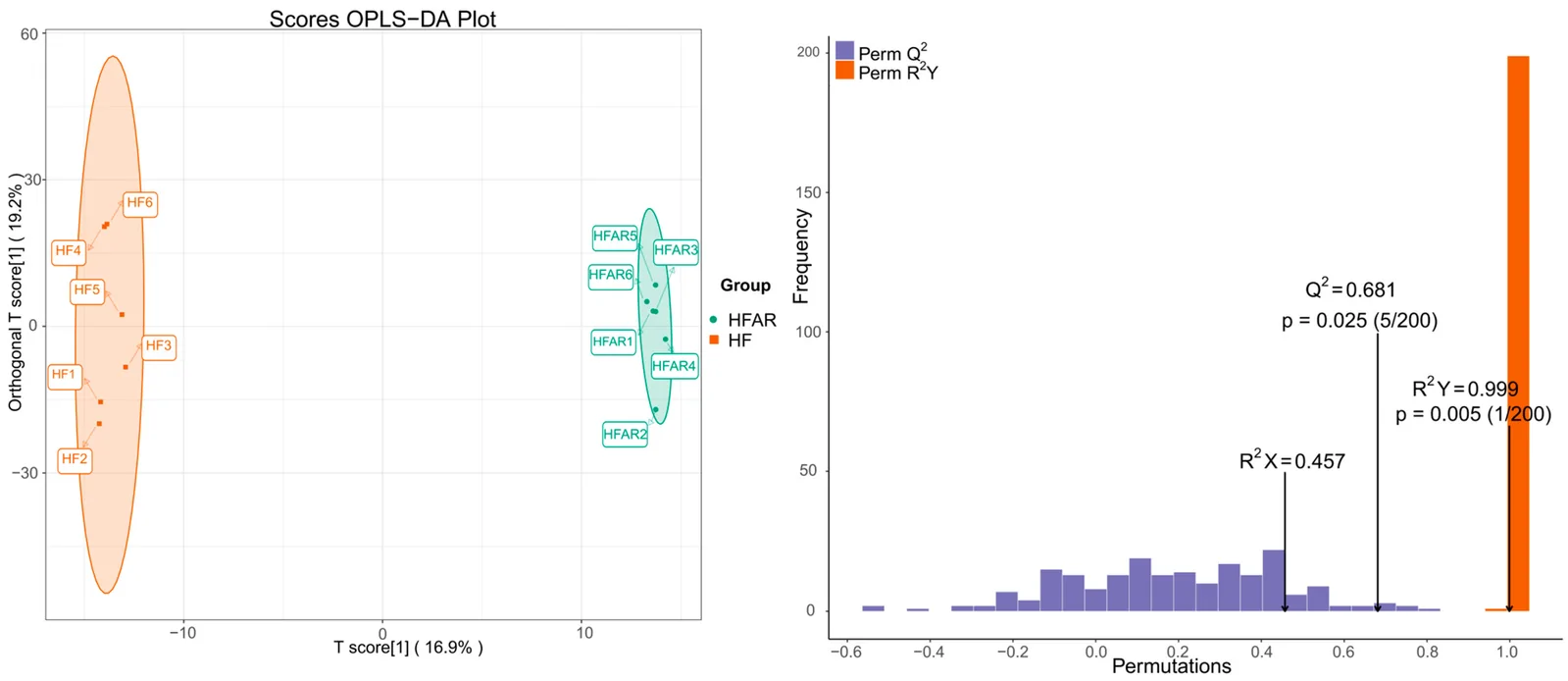
Score plot comparing the High-Fat Diet (HF, orange) and the high-fat diet plus combined aerobic and resistance exercise group. Permutation test for the HF vs. HFAR model.

Untargeted LC–MS-based metabolomic analysis was performed on liver samples collected from the three experimental groups. Unsupervised principal component analysis (PCA) was initially performed to visualize overall metabolic variation and assess data quality. The QC samples clustered tightly on the PCA score plot (Figure 5), indicating high instrument stability and reproducibility. The PCA score plot revealed a clear separation between the C and HF groups along the PC1 axis, whereas the HFAR group exhibited a distinct shift toward the C group, suggesting that exercise intervention partially restored the HFD-disrupted metabolic profile. Orthogonal partial least squares-discriminant analysis (OPLS-DA) demonstrated clear separation between the C and HF groups (Q^2^ = 0.872, R^2^Y = 0.992) and between the HF and HFAR groups (Q^2^ = 0.681, R^2^Y = 0.999), indicating marked alterations in hepatic metabolomic profiles. Permutation tests (200 permutations) confirmed the robustness of these models, with Q^2^ intercepts of 0.15 and −0.05, respectively (both *p* < 0.05), indicating that the observed group separation was unlikely to occur by chance (Figure 6 and Figure 7).

**Figure 8:**
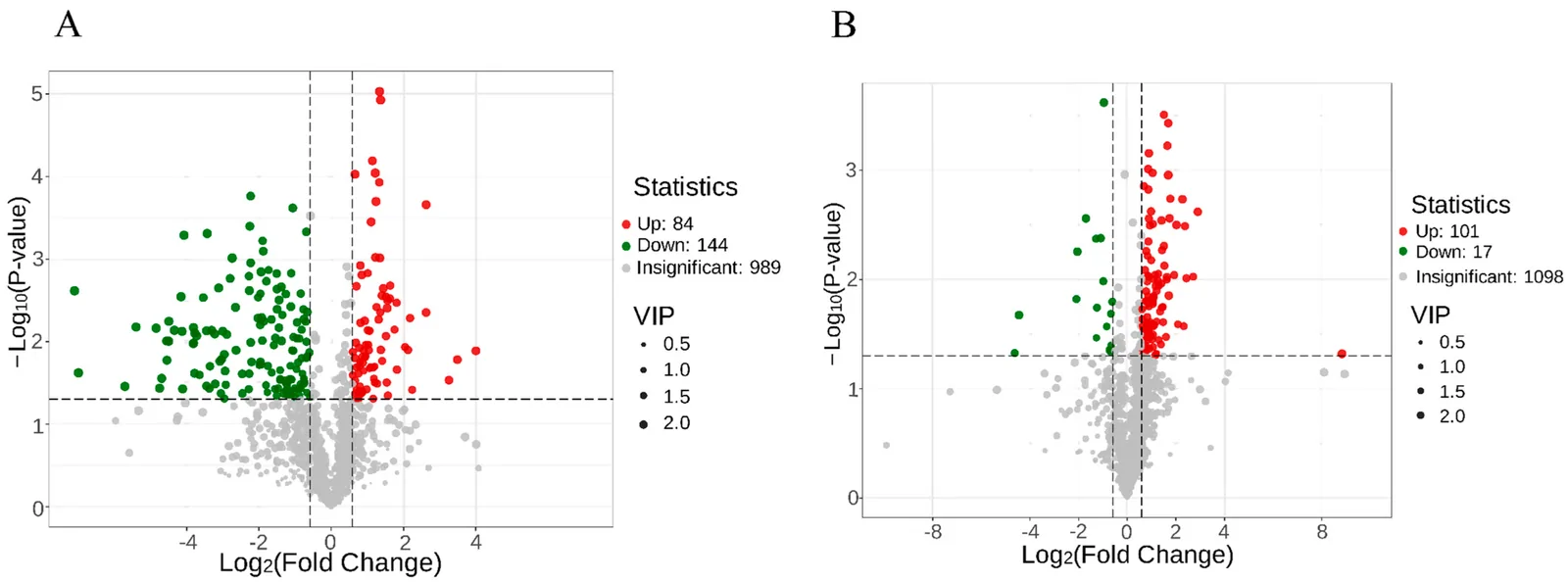
Identification of differential metabolites based on VIP values and statistical significance. (**A**) Volcano plot of differential metabolites in the HF vs. C comparison. (**B**) Volcano plot of differential metabolites in the HFAR vs. HF comparison.

Differentially expressed metabolites were identified using the OPLS-DA model according to the following criteria: variable importance in projection (VIP) > 1, Student’s *t*-test *p* < 0.05, and a fold change ≥ 1.2 or ≤0.83. Compared with the C group, the HF group exhibited 228 differentially expressed metabolites, including 84 upregulated and 144 downregulated metabolites. In comparison, 118 differentially expressed metabolites were identified between the HF and HFAR groups, comprising 101 upregulated and 17 downregulated metabolites (Figure 8). The full list of these differentially expressed metabolites with annotation and statistical parameters is provided in Supplementary Table S1.

**Figure 9:**
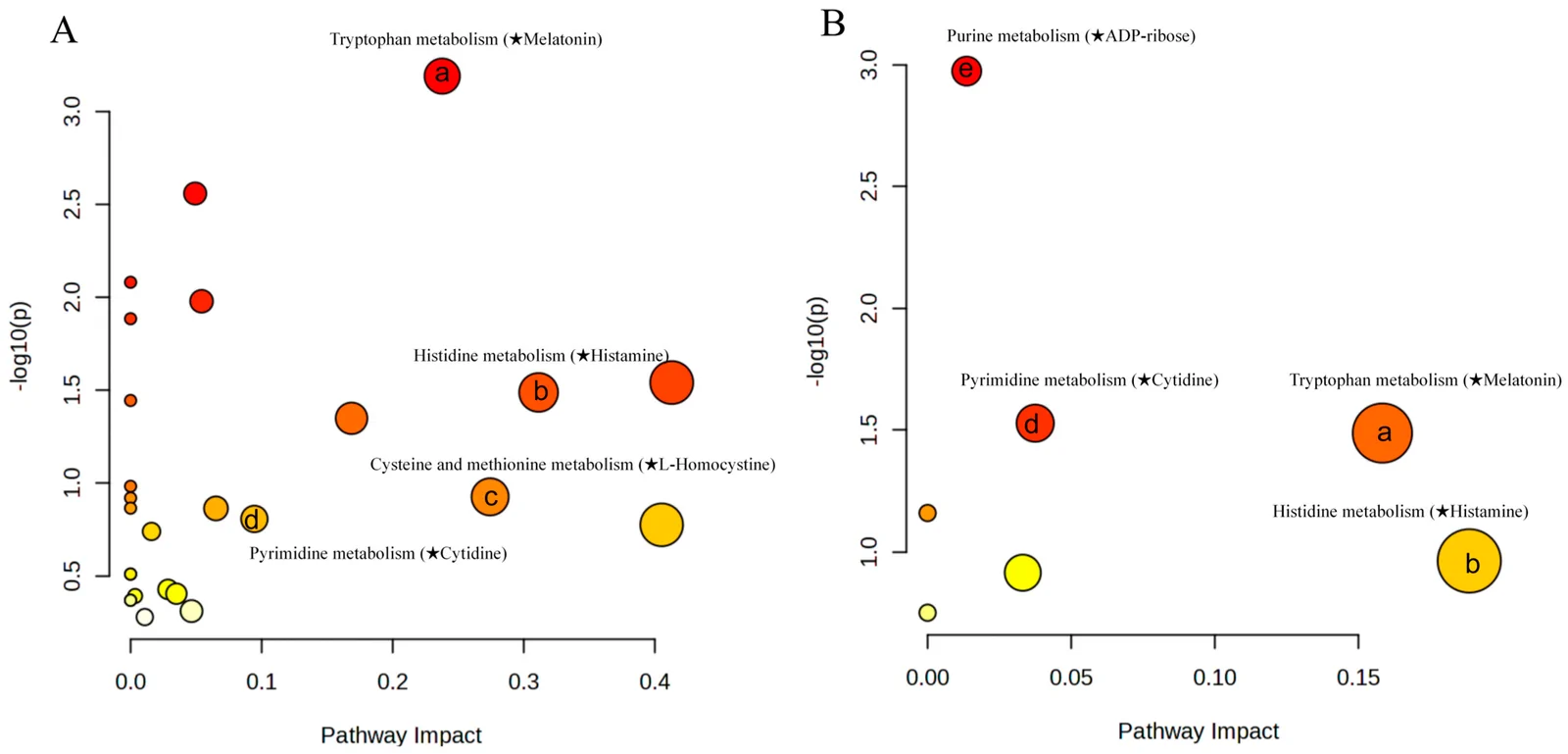
Metabolite and pathway analysis of differential metabolites. (**A**) Pathway enrichment analysis for the HF vs. C group. (**B**) Pathway enrichment analysis for the HFAR vs. HF group. Key pathways harboring core metabolites are marked with lowercase letters: (a) Tryptophan metabolism (Melatonin); (b) Histidine metabolism (Histamine); (c) Cysteine and methionine metabolism (L-Homocystine); (d) Pyrimidine metabolism (Cytidine); and (e) Purine metabolism (ADP-ribose).

Metabolic pathway enrichment analysis of high-confidence endogenous metabolites (MSI Levels 1–2) revealed distinct alterations across groups (Figure 9A,B). In the C vs. HF comparison (Figure 9A), significantly perturbed pathways included tryptophan metabolism, starch and sucrose metabolism, ascorbate and aldarate metabolism, histidine metabolism, cysteine and methionine metabolism, and pyrimidine metabolism. In the HF vs. HFAR comparison (Figure 9B), perturbed pathways narrowed to tryptophan metabolism, histidine metabolism, glycerophospholipid metabolism, purine metabolism, and pyrimidine metabolism, suggesting a partial restoration of metabolic homeostasis following exercise intervention.

**Figure 10:**
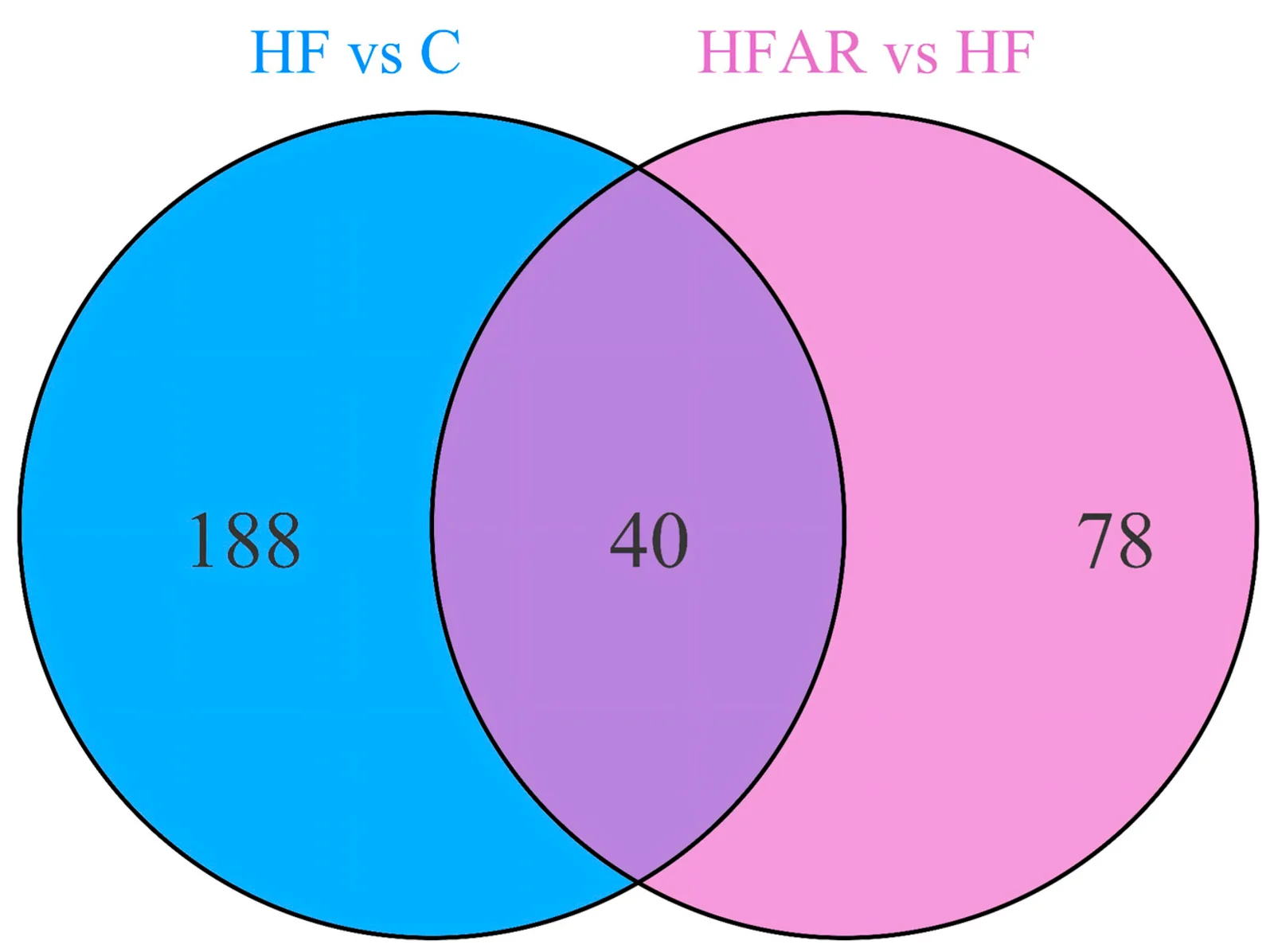
Venn diagram of differentially expressed metabolites.

To pinpoint core metabolic features, we examined the overlap between the two comparative datasets, identifying 40 overlapping endogenous metabolites (Figure 10). From this refined pool, we therefore focused our analysis on six core metabolites that fulfilled all stringent selection criteria (Table 2). Quantitative peak-area data for these six metabolites across all samples are summarized in Supplementary Table S2, and their MSI confidence-level assignments and identification evidence are detailed in Supplementary Table S3. These six core metabolites were subsequently mapped back to the enriched pathways and represented key components of the alterations in pyrimidine, purine, tryptophan, and histidine metabolism: Cytidine and ADP-ribose were mapped to pyrimidine and purine metabolism; Melatonin and Histamine were mapped to tryptophan and histidine metabolism; L-Homocystine was mapped to cysteine and methionine metabolism. Notably, Hexanoylglycine, while assigned as MSI Level 1a, was not mapped to any KEGG pathway, consistent with its classification as a specialized fatty acid-derived metabolite.

### 3.5. Correlation Analysis

**Figure 11:**
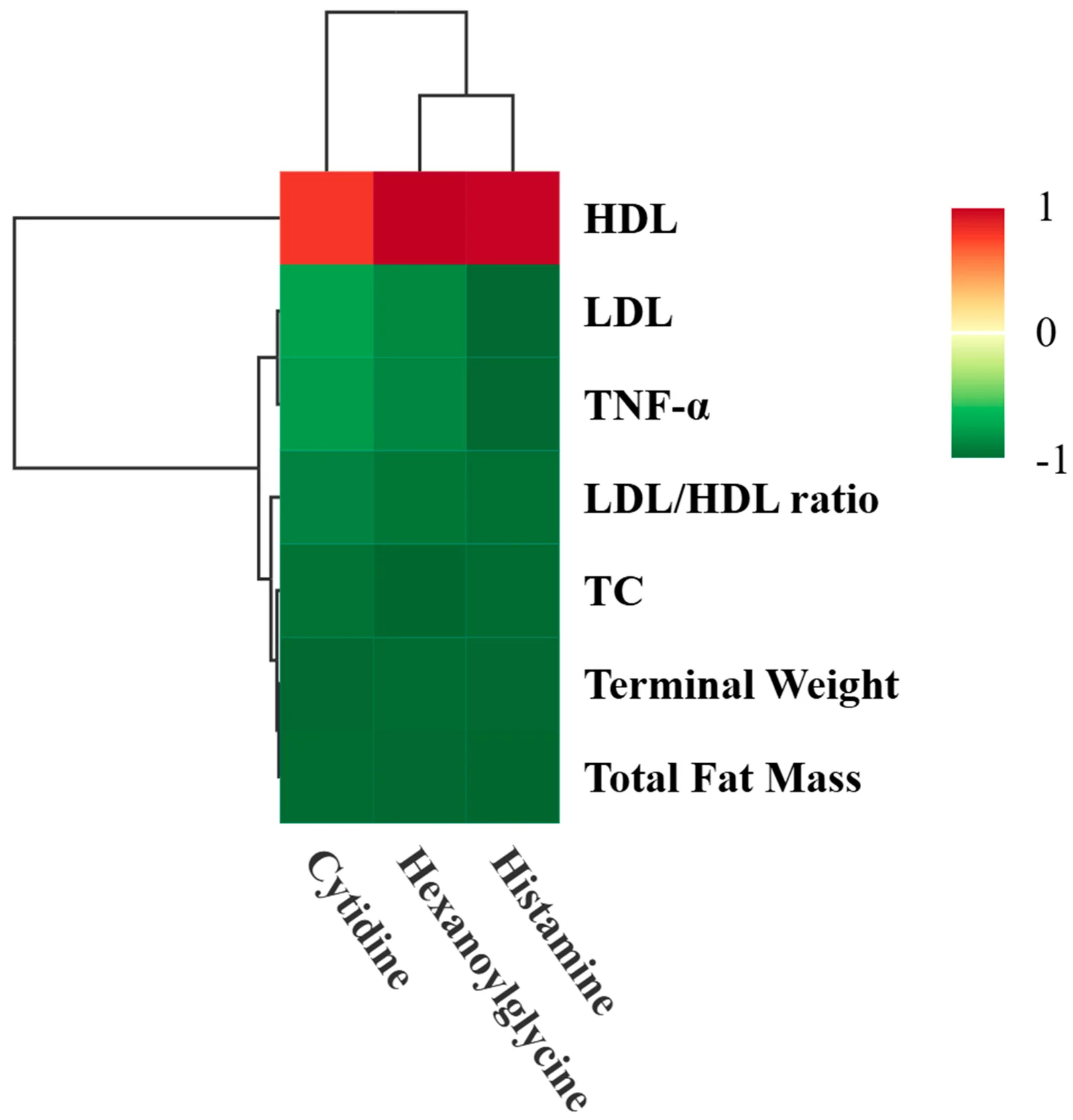
Exploratory correlation heatmap between core hepatic metabolites and physiological phenotypes. The color scale represents the Spearman’s rank correlation coefficient, where red indicates positive correlation and green indicates negative correlation. The intensity of the color reflects the strength of the correlation. Note: Only metabolite–phenotype pairs meeting the pre-defined exploratory screening criteria (|Spearman’s rho| ≥ 0.80 and *p* < 0.10) are displayed.

To further elucidate the systemic impact of the identified hepatic metabolic shifts, we performed an exploratory correlation analysis between the core metabolites and key physiological parameters (Figure 11). Given the limited sample size, we applied a stringent screening criterion (Spearman’s |r| ≥ 0.80 and *p* < 0.10) to highlight the most robust trends for visualization. Under this criterion, three core metabolites (Cytidine, Hexanoylglycine, and Histamine) exhibited strong inverse correlations with markers of obesity, specifically TC, LDL, and Total Fat Mass, and positive associations with HDL. The other three core metabolites (ADP-ribose, L-Homocystine, and Melatonin) did not meet the |r| ≥ 0.80 threshold and are therefore not displayed. To ensure complete transparency, the complete correlation matrix for all six metabolites, including non-significant results, is provided in Supplementary Table S5. No FDR correction was applied to this exploratory analysis; therefore, these correlational findings are hypothesis-generating and require validation in larger cohorts.

## 4. Discussion

Obesity and the associated disturbances in hepatic lipid metabolism have become major global health concerns. The increasing prevalence of metabolic dysfunction-associated steatotic liver disease (MASLD) is closely associated with long-term consumption of a high-fat diet (HFD). Exercise is a well-established nonpharmacological intervention for obesity and obesity-related metabolic disorders, exerting beneficial effects through physiological adaptations to repeated exercise stimuli. Combined aerobic and resistance exercise was selected as a clinically relevant intervention for MASLD based on its documented efficacy in prior literature [8]. Recent evidence suggests that these hepatoprotective effects of exercise are embedded within broader systemic adaptations [17]. In the present study, mice with high-fat-induced obesity underwent an 8-week combined aerobic and resistance exercise intervention. Untargeted metabolomic analysis identified 40 overlapping differentially expressed metabolites, from which six potential core differential metabolites were selected. Pathway enrichment analysis highlighted histidine and pyrimidine metabolism as the primary pathways associated with histidine and pyrimidine metabolism. Given the limited sample size, we applied a stringent screening criterion (Spearman’s |r| ≥ 0.80 and *p* < 0.10) to highlight the most robust trends, which revealed that the core metabolites exhibited inverse associations with pro-steatotic markers (TC, LDL, and total fat mass) and positive associations with the protective HDL. Collectively, these findings indicate that combined aerobic and resistance exercise is associated with attenuated HFD-induced hepatic steatosis and inflammation, potentially involving the modulation of specific hepatic metabolic nodes, particularly within histidine and pyrimidine pathways. Notably, these hepatic benefits occurred in the context of no significant body weight reduction.

The combined aerobic and resistance exercise was associated with significant improvements in the biochemical, histopathological, and morphological indicators of HFD-induced liver injury. Following the 8-week intervention, hepatic levels of ALT, AST, TC, TG, and LDL, and the LDL-C/HDL-C ratio were significantly reduced in the HFAR group compared with the HF group, whereas HDL-C levels were significantly increased. Histological analyses using H&E and Oil Red O staining further demonstrated attenuation of hepatic steatosis, reduced lipid accumulation, and diminished inflammatory cell infiltration following the exercise intervention. In addition, hepatic concentrations of IL-6, IL-1β, and TNF-α were significantly decreased, indicating that combined aerobic and resistance exercise is associated with attenuated HFD-induced hepatic inflammation.

Notably, although liver weight was significantly reduced in the HFAR group compared with the HF group, no significant differences were observed in final body weight, fat mass, or body fat percentage between the two groups. This dissociation between hepatic adaptation and systemic adiposity aligns with a growing body of evidence showing that exercise is associated with improvements in biomarkers of hepatic steatosis, inflammation, and fibrosis even in the absence of substantial weight loss, with improvements reported despite body weight reductions of less than 3% [18,19]. Collectively, these findings suggest that the hepatic benefits of exercise are associated with the modulation of specific hepatic metabolic pathways, and may occur independently of significant changes in systemic adiposity.

Non-targeted metabolomics studies have consistently shown that exercise is associated with sample- and tissue-specific metabolic reprogramming in NAFLD (now termed MASLD), even in the absence of dietary changes or significant weight loss [20]. Metabolomic analysis revealed that eight metabolic pathways were enriched in both the C versus HF and HF versus HFAR comparisons. However, applying our stringent, multi-tiered selection criteria (detailed in Section 2.6) to prioritize robust, exercise-responsive features, the observed perturbations converged specifically on histidine and pyrimidine metabolism, suggesting that these pathways may be associated with the beneficial effects of combined aerobic and resistance exercise on HFD-induced metabolic disturbances.

Among the enriched pathways, histidine metabolism (C vs. HF: −log10(p) = 1.49, Impact = 0.31) emerged as a central node linking exercise to hepatic metabolic remodeling and was associated with alterations in hepatic histamine levels. Histamine, one of the key metabolites identified in this study, is generated from histidine by histidine decarboxylase (HDC) and serves as an important endogenous biogenic amine involved in the regulation of hepatic metabolism. Through activation of four G protein-coupled receptors (H1R–H4R), histamine participates in the regulation of cell proliferation, vascular permeability, tissue repair, inflammatory responses, and immune function [21]. In the present study, hepatic histamine levels were significantly reduced in the HF group and were restored following exercise intervention. A possible hypothesis is that a high-fat diet may suppress hepatic HDC expression or enzymatic activity, potentially limiting histamine biosynthesis and reducing hepatic histamine availability. Based on previous studies, reduced histamine signaling has been proposed to impair H1R-dependent oleoylethanolamide (OEA) synthesis, attenuate peroxisome proliferator-activated receptor-α (PPAR-α)-mediated fatty acid oxidation, and promote hepatic lipid accumulation and inflammation [22]. However, the protein and gene expression levels of HDC, OEA, and PPAR-α were not measured in this study. Therefore, these mechanistic interpretations remain hypothetical and require further validation.

Following the combined exercise intervention, hepatic histamine levels were significantly restored. Previous studies have reported that both aerobic exercise and resistance training increase circulating histamine concentrations [23], potentially leading to activation of H1R- and H2R-mediated signaling pathways. Based on previous literature, it has been proposed that combined aerobic and resistance exercise may enhance hepatic histamine production, which could potentially promote OEA synthesis, stimulate PPAR-α-dependent fatty acid oxidation, and improve hepatic lipid metabolism. This interpretation is supported by the correlation analysis: it exhibited inverse trends with pro-steatotic/pro-inflammatory markers (LDL-C, TNF-α, the LDL/HDL ratio, TC), terminal body weight, and total fat mass, while displaying a positive trend with hepatoprotective HDL-C (|r| ≥ 0.80 and *p* < 0.10). These patterns mirrored the intergroup phenotypic differences (e.g., lower body weight, reduced fat mass, and improved lipid profiles in the HFAR group relative to the HF group). Crucially, despite these consistent directional trends, no significant differences were observed between the HF group and HFAR group in final body weight and total fat mass, suggesting that restored histidine metabolism may reflect a hepatic adaptive response to exercise, independent of significant changes in systemic adiposity.

Cytidine, a pyrimidine nucleoside, was markedly reduced in the livers of mice in the HF group, and this reduction was significantly reversed by combined aerobic and resistance exercise intervention. In metabolite pathway analysis, the enriched pyrimidine metabolism pathway exhibited changes consistent with those of cytidine, suggesting that cytidine, as one of the potential core metabolites identified in this study, is associated with the regulation of hepatic metabolism by combined exercise through the pyrimidine metabolism pathway. This observation is consistent with findings from a study in ob/ob mice, which showed that disrupted pyrimidine metabolism is closely associated with hepatic lipid accumulation [24]. As a key substrate of the pyrimidine salvage pathway, cytidine may reflect adaptive alterations in hepatic nucleotide metabolism through changes in its abundance. Studies have shown that hepatic pyrimidine levels are markedly reduced in models of NAFLD (now termed MASLD) [25]. This is consistent with the decreased hepatic cytidine levels observed in the HF group in the present study. The restoration of cytidine levels in the HFAR group may be associated with improved hepatic lipid metabolic, potentially through promoting pyrimidine nucleotide synthesis. It has been hypothesized that exercise-induced metabolic stress may lead to activation of the hepatic pyrimidine salvage pathway to support hepatocytes in maintaining nucleotide pool homeostasis, thereby ensuring RNA synthesis and cellular functional recovery; however, this remains speculative and requires further validation. Further correlation analysis revealed that hepatic cytidine levels tended to be negatively correlated with final body weight, total fat mass, TNF-α, and hepatic lipid deposition indicators (LDL-C, TC), and positively correlated with HDL-C. Together with the finding that final body weight and total fat mass did not decrease significantly, these correlational trends suggest that the restoration of cytidine levels may be associated with the regulation of hepatic lipid metabolism, independent of significant changes in body weight and adiposity. In other words, the restoration of hepatic cytidine levels was associated with the hepatoprotective effects of combined exercise, potentially through the modulation of the pyrimidine metabolism pathway; however, this remains a hypothesis-generating observation requiring further validation.

Apart from cytidine and histamine, the changes in the remaining potential core differential metabolites further suggest that the hepatic metabolic network underwent remodeling. ADP-ribose was significantly decreased in the HF group and significantly increased in the HFAR group compared with the HF group. This change is consistent with the finding that purine metabolism was enriched only in the HF vs. HFAR comparison, suggesting that exercise may be associated with partial normalization of purine/energy metabolism induced by a high-fat diet. L-homocystine returned toward control levels after exercise intervention, which is consistent with the enrichment of cysteine and methionine metabolism only in the C vs. HF comparison, indicating that the dysregulation of this pathway may have been driven by the high-fat diet stage and showed a trend toward restoration after exercise. Tryptophan metabolism was enriched in both the C vs. HF and HF vs. HFAR comparisons, and its downstream metabolite melatonin was markedly restored in the HFAR group, suggesting that combined exercise may be associated with partial restoration of the tryptophan metabolic disorder induced by a high-fat diet. In addition, although hexanoylglycine was not annotated to any classical KEGG pathway, it was decreased in the HF group and increased in the HFAR group, and exhibited correlation trends similar to those of cytidine and histamine. In contrast, no such correlation trends were observed for ADP-ribose, L-homocystine, or melatonin. Collectively, the directional changes in these metabolites suggest that exercise intervention is associated with the coordinated remodeling of multiple metabolic pathways.

The hepatic metabolic features described above are consistent with the skeletal muscle and serum metabolic changes recently reported by our group [11]. While our previous study focused on the gut-muscle axis, the present findings extend these observations to the liver, suggesting that combined exercise is associated with coordinated metabolic adaptations across multiple tissues. Notably, these hepatic benefits occurred without significant reductions in body weight, suggesting a potential pattern of weight-independent hepatoprotection.

Several limitations of the present study should be acknowledged. First, the study employed a modeling protocol involving 8 weeks of high-fat feeding combined with 8 weeks of exercise intervention in C57BL/6J mice, which primarily mimics the early stage of metabolic dysfunction-associated steatotic liver disease (MASLD). Therefore, caution is warranted when extrapolating these findings to advanced metabolic dysfunction-associated steatohepatitis (MASH) or to the complex pathophysiological context of human disease. Second, the hepatic metabolomic analysis represents a static snapshot at the end of the intervention and lacks longitudinal time-series data, making it difficult to establish the causal temporal sequence of changes in key metabolites. Third, due to the limited sample size (n = 6) and the exploratory nature of this study, no FDR correction was applied to the differential metabolite screening or pathway enrichment analysis. All six core metabolites had FDR > 0.05; therefore, these findings should be considered exploratory and hypothesis-generating. Furthermore, although the six core metabolites were confidently identified using authentic standards (MSI Level 1a/1b) and the corresponding MS/MS mirror plots were provided (Supplementary Figure S1), absolute quantification was not performed, and the protein expression levels of key metabolic enzymes were not validated by qPCR or Western blotting. The precise metabolic regulatory networks of certain metabolites (e.g., hexanoylglycine) in mammals remain to be elucidated. Finally, the study design did not include aerobic-only, resistance-only, or pair-fed control groups; therefore, the specific contributions of the aerobic and resistance components, as well as the potential confounding effects of caloric intake, were not evaluated. Additionally, individual activity levels and accurate food efficiency were not assessed, which limits the interpretation of energy balance.

## 5. Conclusions

Combined aerobic and resistance exercise was associated with marked hepatic metabolic reprogramming. At the mechanistic level, this study highlighted the pivotal associations of histidine and pyrimidine metabolism and identified six metabolites (MSI Level 1a/1b), including histamine, cytidine, and hexanoylglycine. At the phenotypic level, this metabolic remodeling was accompanied by significant pathological improvements: without a significant reduction in terminal body weight, exercise was associated with attenuated hepatic lipid deposition, lower hepatic transaminase levels, and reduced hepatic inflammation. These findings provide valuable insights into the interplay between exercise and hepatic metabolism, and support the notion of weight-independent hepatoprotection. Among these metabolites, specific molecules such as hexanoylglycine hold promise as potential biomarkers for evaluating the efficacy of exercise interventions, offering new insights for optimizing non-pharmacological intervention strategies for obesity-related MASLD.

## Figures and Tables

**Table 1:** Formal Exercise Training Protocol.

Training Period	Aerobic Training	Resistance Training
Week 1	Treadmill running at 12 m/min for 30 min	Ladder climbing with a load equivalent to 10% of body weight
Weeks 2–3	Treadmill running 12 m/min for 45 min	Ladder climbing with a load equivalent to 30% of body weight
Weeks 4–5	Treadmill running at 15 m/min for 45 min	Ladder climbing with a load equivalent to 50% of body weight
Weeks 6–8	Treadmill running at 15 m/min for 60 min	Ladder climbing with a load equivalent to 70% of body weight

**Table 2:** Potential core Differential Metabolites Identified.

Compounds	MSI Level	Class	C vs. HF				HF vs. HFAR			
			VIP	FDR	FC	Trend (No-Minal *p*)	VIP	FDR	FC	Trend (No-Minal *p*)
Cytidine	1a	Nucleotide and Its metabolites	1.7700	0.0899	0.2455	↓ **	1.7724	0.2587	2.4691	↑ *
Hexanoylglycine	1a	Amino acid derivatives	1.8436	0.1092	0.3856	↓ **	1.8536	0.3127	1.7674	↑ *
ADP-ribose	1b	Nucleotide and Its metabolites	1.4604	0.1841	0.4131	↓ *	1.5860	0.3733	2.0954	↑ *
L-Homocystine	1b	Amino acids	1.4695	0.1420	2.6579	↑ *	1.7668	0.2482	0.5047	↓ *
Melatonin	1b	Hormones and hormone related compounds	1.4207	0.2073	4.1256	↑ *	1.3398	0.3493	0.4151	↓ *
Histamine	1b	Amino acid derivatives	1.9087	0.1409	0.0427	↓ *	1.4518	0.3461	1.9769	↑ *

Note: *: *p* < 0.05; **: *p* < 0.01. MSI Level 1a (Total.score > 0.90) and Level 1b (0.50 < Total.score < 0.90) were assigned based on matches to authentic standards (Standard, MS1, RT, MS2) in the service provider’s in-house database (MWDB). Total.scores: Cytidine (0.94), Hexanoylglycine (0.93), ADP-ribose (0.75), L-Homocystine (0.73), Melatonin (0.75), Histamine (0.67). The corresponding MS/MS mirror plots and standard CAS numbers are provided in Supplementary Figure S1 and Supplementary Table S4, respectively.

## Data Availability

The metabolomics data generated in this study have been deposited in the MetaboLights repository under accession number: MTBLS15011. Available at: https://www.ebi.ac.uk/metabolights/MTBLS15011 (accessed on 7 October 2026).

## Supplementary Materials

The following supporting information can be downloaded at https://www.mdpi.com/article/10.3390/metabo16100761/s1. Supplementary Table S1. Full list of differentially expressed metabolites identified by OPLS-DA (VIP > 1.0, Student’s *t*-test *p* < 0.05, fold change ≥ 1.2 or ≤0.83) in the HF vs. C and HFAR vs. HF comparisons, including annotation, VIP, *p*-value, FDR, Log_2_FC, and trend. Supplementary Table S2. Chromatographic–mass spectrometric parameters and sample-wise peak-area quantitation (C/HF/HFAR, *n* = 6 each; QC, *n* = 3) for the six core metabolites. Supplementary Table S3. MSI confidence-level assignments and identification evidence for the six core metabolites, with notes on mass-error tolerance and Level 1a/1b classification. Supplementary Table S4. Standard information (CAS numbers, catalog numbers) for the six core metabolites. Supplementary Table S5. Complete correlation matrix for all six core metabolites. Supplementary Figure S1. MS/MS mirror plots of the six core metabolites.

## Abbreviations

The following abbreviations are used in this manuscript:

ALT	Alanine aminotransferase
AST	Aspartate aminotransferase
C	Control group
ELISA	Enzyme-linked immunosorbent assay
H&E	Hematoxylin and eosin
HDC	Histidine decarboxylase
HDL-C	High-density lipoprotein cholesterol
HF	High-fat diet group
HFAR	High-fat diet plus combined aerobic and resistance exercise group
HFD	High-fat diet
IL	Interleukin
IQR	Interquartile range
KNN	K-nearest neighbors
LC-MS	Liquid chromatography–mass spectrometry
LDL-C	Low-density lipoprotein cholesterol
MASLD	Metabolic dysfunction-associated steatotic liver disease
NAFLD	Nonalcoholic fatty liver disease
OEA	Oleoylethanolamide
OPLS-DA	Orthogonal partial least squares-discriminant analysis
PPAR-α	Peroxisome proliferator-activated receptor-α
QC	Quality control
SD	Standard deviation
SPF	Specific pathogen-free
SVR	Support vector regression
TC	Total cholesterol
TG	Triglycerides
TNF-α	Tumor necrosis factor-alpha
UHPLC	Ultra-high-performance liquid chromatography
VIP	Variable importance in projection
WAT%	Percentage of white adipose tissue
